# 基于磁性氧化石墨烯核酸适配体分离材料构建17*β*-雌二醇传感器

**DOI:** 10.3724/SP.J.1123.2024.06009

**Published:** 2025-01-08

**Authors:** Xinyu JIN, Leyuan CHEN, Yanna LIU, Wenjing XIE, Hanyong PENG

**Affiliations:** 1.中国科学院生态环境研究中心,环境化学与生态毒理学国家重点实验室, 北京 100085; 1. State Key Laboratory of Environmental Chemistry and Ecotoxicology, Research Center for Eco-Environmental Sciences, Chinese Academy of Sciences, Beijing 100085, China; 2.中国科学院大学, 北京 100049; 2. University of Chinese Academy of Sciences, Beijing 100049, China; 3.中国科学院大学杭州高等研究院,环境学院, 浙江 杭州 310024; 3. School of Environment, Hangzhou Institute for Advanced Study, University of Chinese Academy of Sciences, Hangzhou 310024, China

**Keywords:** 17*β*-雌二醇, 磁固相分离, 荧光生物传感器, 磁性氧化石墨烯, 17*β*-estradiol (E2), magnetic solid phase separation, fluorescence biosensor, magnetic graphene oxide (MGO)

## Abstract

17*β*-雌二醇(E2)是天然的甾体雌激素,在生物体的各项生理活动中发挥着至关重要的作用。然而,外源性E2也被归类为内分泌干扰物(EDC),即使在ng/L水平上也会干扰内分泌系统功能,且在医疗、畜牧业废水中均能检测到E2污染。目前,E2的检测方法主要以色谱-质谱联用方法为主,但受仪器等限制,难以应用于现场检测或大量样品检测。为了解决该难题,本工作用油包水微乳液法(W/O microemulsion)合成了一种基于磁性氧化石墨烯(MGO)的核酸分离材料,并进行了系统的表征,以开发高灵敏度、快速检测、高通量的荧光恢复(“turn-on ”)型生物传感器,用于检测环境样品中的E2。传感器基于荧光共振能量转移原理(FRET),即MGO通过吸附荧光基团标记的E2核酸适配体发生FRET效应猝灭荧光信号,而在E2存在时,核酸适配体与E2结合,从MGO上脱附,实现荧光信号恢复,且信号响应与一定范围的E2质量浓度呈线性关系。本工作引入了磁性固相分离方法,降低了背景信号值,从而提高了方法的灵敏度。本方法中相对荧光恢复强度与E2质量浓度呈良好的线性关系,检出限低至1 ng/mL,对多种干扰离子有较好的抵抗性,且在同族雌激素类似物中有良好的选择性。与色谱-质谱检测方法相比,该方法检测时间短,成本低,操作简便,可应用于环境水样中E2的快速检测。

## Abstract (en)

17*β*-Estradiol (E2) is a natural steroidal estrogen essential for a variety of physiological functions in organisms. However, external E2, which is renowned for its potent biological effects, is also considered to be an endocrine-disrupting compound (EDC) capable of disturbing the normal operation of the endocrine system, even at nanogram-per-liter (ng/L) concentrations. Studies have revealed that medical and livestock wastewater can be contaminated with E2, which poses potential risks to human health. Currently, the primary method for detecting E2 relies on liquid chromatography-mass spectrometry, which is limited with regard to on-site or large-scale sample testing due to instrumental constraints. Herein, we developed a magnetic graphene oxide (MGO)/aptamer separating material. The MGO was synthesized by creating a water-in-oil microemulsion at 90 ℃, an agarose hydrogel to load the Fe_3_O_4_ nanoparticles, and layered graphene oxide (GO). In contrast to conventional methods, such as chemical co-precipitation and solvothermal approaches, this method is more time-efficient and does not require high temperature or pressure. Moreover, the use of a physical encapsulation technique for enwrapping the Fe_3_O_4_ nanoparticles and layered GO eliminates the need for chemical modification. This approach reduces the use of harmful chemicals, ensures complete loading, and results in highly efficient encapsulation. The MGO was characterized using Fourier-transform infrared (FT-IR) spectroscopy and transmission electron microscopy (TEM), as well as dynamic light scattering (DLS) and Zeta potential analyses, which revealed that the Fe_3_O_4_ nanoparticles had been successfully loaded onto the GO to produce MGO particles mainly around 5 μm in size. Additionally, this study demonstrated that the aqueous MGO dispersion is highly stable.

This substance was used to develop a fluorescent biosensor that uses a “turn-on” mechanism to rapidly and highly sensitively detect E2. MGO is capable of adsorbing a fluorescently labeled E2 aptamer (FAM-Apt) in solution, resulting in fluorescence quenching through fluorescence resonance energy transfer (FRET) between the fluorescent group and graphene. However, E2 preferentially binds to FAM-Apt, resulting in the FAM-Apt separating from the MGO in the presence of E2, thereby restoring fluorescence. The developed biosensor exhibits a robust linear correlation between relative fluorescence intensity and E2 mass concentration in the 1-1000 ng/mL range, and boasts a low detection threshold of 1 ng/mL. The use of MGO as an absorbent and fluorescence quencher led to an E2-detection limit that is two orders of magnitude lower than that of a GO-based sensor. This biosensor also outperforms other aptamer-based systems in terms of detection time, linear range, and sensitivity; it also demonstrates remarkable resilience toward various interfering ions and exhibits strong selectivity among structurally similar estrogen analogs. A range of ions commonly present in water samples were introduced into the reaction system at specific concentrations to gauge the impact of interfering ions on sensor performance. With the exception of Fe^3+^ ions at 0.3 mg/L, which led to a lower fluorescence intensity, interfering ions were found to exhibit minimal effects. Biosensor specificity and selectivity were further scrutinized by introducing four estrogenic disruptors, including estriol (E3), 17*β*-ethynylestradiol (EE2), estrone (E1), and bisphenol A (BPA), each at a mass concentration of 1 μg/mL under the same reaction conditions used to detect E2. The recovered relative fluorescence-signal values for E1 and E3 were determined to be 33% and 23% that of E2, respectively, while EE2 and BPA hardly elicited any fluorescence signal recovery, thereby highlighting the ability of the biosensor to precisely detect E2 with minimal interference from estrogen analogs. The efficacy of the MGO-FAM-Apt biosensor was subsequently validated by testing river-water samples containing known quantities of added E2, which yielded recoveries of between 91.0% and 110.0%, thereby affirming the reliability of this biosensor for use in practical applications. The developed sensor may be somewhat limited compared to liquid chromatography-high-resolution mass spectrometry in detection limit, but the developed biosensor is cost-effective, detects rapidly, and is capable of simultaneously analyzing multiple samples, making it suitable for on-site or large-scale E2 testing of environmental water samples.

17*β*-雌二醇(E2)作为天然的固醇类激素,在调节生殖功能和第二性征发育、维持钙稳态和维护心脑血管系统健康等方面有诸多功能^[1]^,但外源E2活性极强,ng/L级别的剂量就足以影响生物的正常生理活动^[2]^。已有多项研究证实外源E2会对多种鱼类的生殖系统及其健康造成不良影响^[3,4]^,对生物甚至人类的繁衍产生影响,从而被归为内分泌干扰化合物。E2可作为活性成分被应用于妇科病的激素疗法或畜牧业中,所以医疗废水^[5]^和牲畜粪便^[6]^是E2污染的重要来源,其对环境水的污染急需重视,建立对环境水样中E2的快速、简便、灵敏检测方法至关重要。

目前E2的常用检测技术主要分为色谱法^[7⇓-9]^、电化学法^[10]^和生物传感器法^[11⇓⇓⇓⇓⇓-17]^。最常用的是色谱法,如高效液相色谱(HPLC)或气相色谱与质谱(MS)联用,具有灵敏度高、检出限低的特点,但由于样品前处理流程复杂、检测仪器设备昂贵且需专业操作人员,难以广泛应用于现场快速检测。生物传感器法具有检测速度快、易于构建高通量分析体系的优点,主要包括免疫分析法和核酸适配体传感器两种类型。免疫分析法采用蛋白抗体作为特异性识别探针,其中抗体成本高、生产周期长,而核酸适配体是具有与抗体类似功能的DNA或RNA分子,具有E2识别特异性高、合成简单、成本低廉、稳定性好等优点^[18]^。然而,基于E2适配体构建的荧光传感器往往在均相体系中反应,较高的荧光背景信号限制了方法的检测灵敏度。

因此,本研究采用改进的微乳液合成法^[19]^,通过琼脂糖凝胶将Fe_3_O_4_纳米磁珠包覆在氧化石墨烯(GO)片层上,得到磁性氧化石墨烯(MGO)。GO可以与核酸通过*π-π*堆积、氢键和静电相互作用等方式结合^[20,21]^,因此具有良好的核酸吸附性能,且有较高的荧光猝灭效率^[22]^。当样品中不含E2时,荧光标记的核酸适配体(FAM-Apt)被吸附在MGO中的GO表面上,荧光猝灭;而当E2存在时,FAM-Apt特异性识别E2,相互作用形成复合物,从GO上解离,并恢复其荧光信号,构建荧光恢复(“turn-on”)型生物传感器。在磁场作用下,体系中吸附在MGO上的过量FAM-Apt通过磁分离从溶液中移除,可降低体系中的背景荧光信号,从而提高方法对低浓度E2的检测灵敏度。本文还将所开发的方法与液相色谱-高分辨质谱(LC-HRMS)方法进行了比较,对比了两种方法的分析性能,并应用于实际样品分析。

## 1 实验部分

### 1.1 仪器与试剂

SpectraMax^®^ iD5多功能酶标仪(美国Molecular Devices); Vanquish Horizon LC-Q Exactive^TM^高分辨质谱仪(用4.1版本的Xcalbur软件进行控制)和Nicolet iN10MX傅里叶变换红外光谱仪(FT-IR,美国Thermo Fisher Science); Zetasizer Nano ZS90纳米粒度及Zeta电位仪(英国马尔文仪器有限公司); Tecnai G2 F30透射电子显微镜(TEM, 美国FEI公司);KQ-700VDE型双频数控超声波清洗器(昆山市超声仪器有限公司); VORTEX-GENIE^®^2涡旋混合仪(美国Scientific Industries); S1010E微型离心机和SCI550-Pro加热磁力搅拌器(美国SCILOGEX); MYP2011-100恒速电动搅拌器(上海梅颖浦仪器仪表制造有限公司);微量移液器(德国Eppendorf); Milli-Q Advantage A10超纯水仪(德国Merck)。

琼脂糖(Gel strength(1% Gel)≥1200 g/cm)、矿物油(CP级)、Span-80(CP级)购于北京索莱宝科技有限公司;Fe_3_O_4_纳米球(15~30 nm,上海阿拉丁生化科技股份有限公司);E2有效成分含量大于99%,购于河北百灵威超精细材料有限公司;5 mg/mL氧化石墨烯分散液(溶剂为水)、双酚A(bisphenol A, BPA,有效成分含量大于99.0%)、雌三醇(estriol, E3)、17*β*-乙炔基雌二醇(17*β*-ethynylestradiol, EE2)、雌酮(estrone, E1)购于江苏先丰纳米材料科技有限公司,E3、EE2和E1有效成分含量均高于98%;六水合氯化镁(BR级)、氯化钾(ACS级)、氯化钠(ACS级)、Trizma^®^ base(ACS级)均购于美国Sigma-Aldrich;盐酸(GR级)购于昆山金城试剂有限公司;二甲基亚砜(DMSO,生物纯级,≥99.7%,美国 Sigma-Aldrich);甲醇(HPLC级)购自比利时Fisher Chemical;无水乙醇(AR级)购于上海泰坦科技股份有限公司;丙酮(AR级)购于太仓沪试试剂有限公司。

本研究所用带羧基荧光素(carboxyfluorescein, FAM)荧光基团修饰的单链DNA适配体(DNA序列为3'- ACGACTTAAGGTATGTGATCTTAGTTGTA GTTCAAGTCGT-5')订购于梓熙生物科技有限公司(以下简称FAM-Apt,以HPLC纯化得到),使用前将粉末溶于无酶水中配制为100 μmol/L的溶液,并逐级稀释至100 nmol/L备用。

### 1.2 MGO材料的制备与表征

采用改进的油包水微乳液法(W/O microemulsion)合成MGO^[19]^。移取100 μL矿物油溶解于2900 μL Span 80中,形成油相混合液;移取450 μL 5 mg/mL Fe_3_O_4_纳米磁珠水溶液、450 μL 5 mg/mL片层GO溶液、100 μL超纯水和20 mg琼脂糖混合溶解得到水相混合液,并超声处理至均匀。将油相混合液加热至90 ℃,同时搅拌并缓慢滴加20 μL无水乙醇;同时将水相混合液预热至90 ℃(升温过程中持续搅拌),缓慢滴加水相混合液至油相混合液中,完全加入后在90 ℃下反应10 min,继续搅拌并冷却至室温,得到MGO粗品。在磁力架上吸附固定MGO粗品,移除液体,然后用4∶1(v/v)的丙酮-水混合洗液洗涤4次,随后用超纯水洗涤4次。弃去洗涤液,加超纯水定容至4 mL,在80 Hz条件下超声15 min,得到分散性良好的MGO水溶液。

将MGO水溶液稀释1000倍,使用超声处理20 min,进行动态光散射(DLS)和Zeta电位测量。通过磁力架对MGO和上清液进行磁性分离,将MGO用无水乙醇洗涤3次,然后在红外灯下干燥,得到MGO粉末样品。将合成原料GO水溶液直接干燥,然后使用FT-IR对MGO和GO的官能团组成进行分析和比对。

### 1.3 MGO-FAM-Apt生物传感器的构建

向PCR管(聚合酶链式反应管)中加入20 μL 5×Buffer(750 mmol/L NaCl、25 mmol/L MgCl_2_、1 mol/L Tris-HCl, pH=9), 10 μL 100 nmol/L FAM-Apt溶液、50 μL超纯水和10 μL E2溶液,在室温下避光孵育10 min后,加入10 μL MGO溶液,混合均匀,避光孵育5 min。随后用磁力架分离MGO磁珠和上清液。分离后将100 μL上清液(传感器反应体系)转移至96孔板中,用酶标仪测量荧光恢复信号值,并计算相对荧光恢复值,计算公式如下:

相对荧光恢复值= $\frac{F_{1}-F_{0}}{F_{0}}$

其中*F*_1_是加入E2后检出的荧光恢复信号值,*F*_0_是用10 μL超纯水替代E2溶液后检出的荧光信号值,即空白样品的荧光背景信号值。

相对荧光恢复值在一定范围内与E2浓度成正比,据此得到相对荧光恢复值-E2质量浓度线性回归方程,用于E2定量。

### 1.4 标准溶液配制、抗干扰能力与特异性检验

为检验传感器的灵敏度,配制E2标准溶液进行检验。E2标准溶液母液质量浓度为1 mg/mL,称取E2标准品用DMSO溶解配制,然后用DMSO稀释至10 μg/mL后用1×Buffer(150 mmol/L NaCl、5 mmol/L MgCl_2_、200 mmol/L Tris-HCl, pH=9)梯度稀释至质量浓度分别为1、5、10、50、100、500、1000、5000、10000 ng/mL。

据GB 3838-2002《地表水环境质量标准》,水样中可能出现干扰离子,在反应体系中分别添加100 mg/L氯化钙(CaCl_2_)、0.3 mg/L氯化铁(FeCl_3_)、0.1 mg/L氯化锰(MnCl_2_)、250 mg/L硫酸钠(Na_2_SO_4_)和10 mg/L硝酸钠(NaNO_3_),检测终质量浓度为10 ng/mL的E2,讨论干扰离子对传感器性能的影响。

为检测传感器的特异性,用质量浓度均为1 μg/mL的4个雌激素类化合物——E3、EE2、E1和BPA,分别如1.3节中步骤反应检测,比较荧光恢复值差异,验证传感器的特异性。

### 1.5 定量分析及水样加标试验

从北京清河中采集河水样品,以0.22 μm滤膜过滤得到澄清液。取10 μL澄清液加入所构建的传感器反应溶液(100 μL)中,以E2质量浓度分别为0、1、5、7.5、10、50、75、100 ng/mL绘制标准曲线,并进行E2质量浓度为1、3、6 ng/mL的加标试验。

## 2 结果与讨论

### 2.1 MGO合成、表征及核酸吸附应用

本工作采用微乳液法合成MGO,在90 ℃下基于油包水微乳液利用琼脂糖水凝胶固定交联Fe_3_O_4_纳米磁珠和片层GO。与化学共沉淀、溶剂热法等传统合成方法^[23,24]^相比,该法所用时间更短,不需要高温高压条件,同时因为采用物理包裹的方法负载磁珠和GO,无需进行化学修饰反应,使用的有毒有害试剂少,且能达到完全负载,包裹效率高。MGO具有良好的分散性以及较高的荧光猝灭率,能够高效吸附核酸。在200 mmol/L Tris-HCl (pH=9)、5 mmol/L MgCl_2_、150 mmol/L NaCl、10 nmol/L FAM-Apt的条件下,MGO材料用于DNA分离的吸附率为97%,且可清洗后重复利用^[25]^。

如图1a所示,通过TEM对MGO的形貌进行表征,发现大量黑色Fe_3_O_4_纳米磁珠分布在褶皱丝状的GO薄片上,证明磁珠被成功负载在GO片层上。DLS和Zeta电位测量表明,MGO尺寸大小主要集中在5 μm, Zeta电位平均值为-8.16 mV,标准差为0.797 mV,具有较好的稳定性。我们对合成的MGO和GO分别进行了FT-IR表征,见图1b,在3400、1650、1100 cm^-1^处有吸收峰,3400 cm^-1^的宽吸收峰由-OH的伸缩振动产生,1650 cm^-1^处的峰来自羧基中C=O的拉伸振动,1100 cm^-1^处的峰是由于C-O-C的振动导致;说明MGO具有丰富的含氧官能团,包括环氧基、羰基、羧基和羟基等^[21]^,可以与核酸分子通过*π-π*堆积、氢键和静电相互作用等方式结合^[20]^。

**图1:**
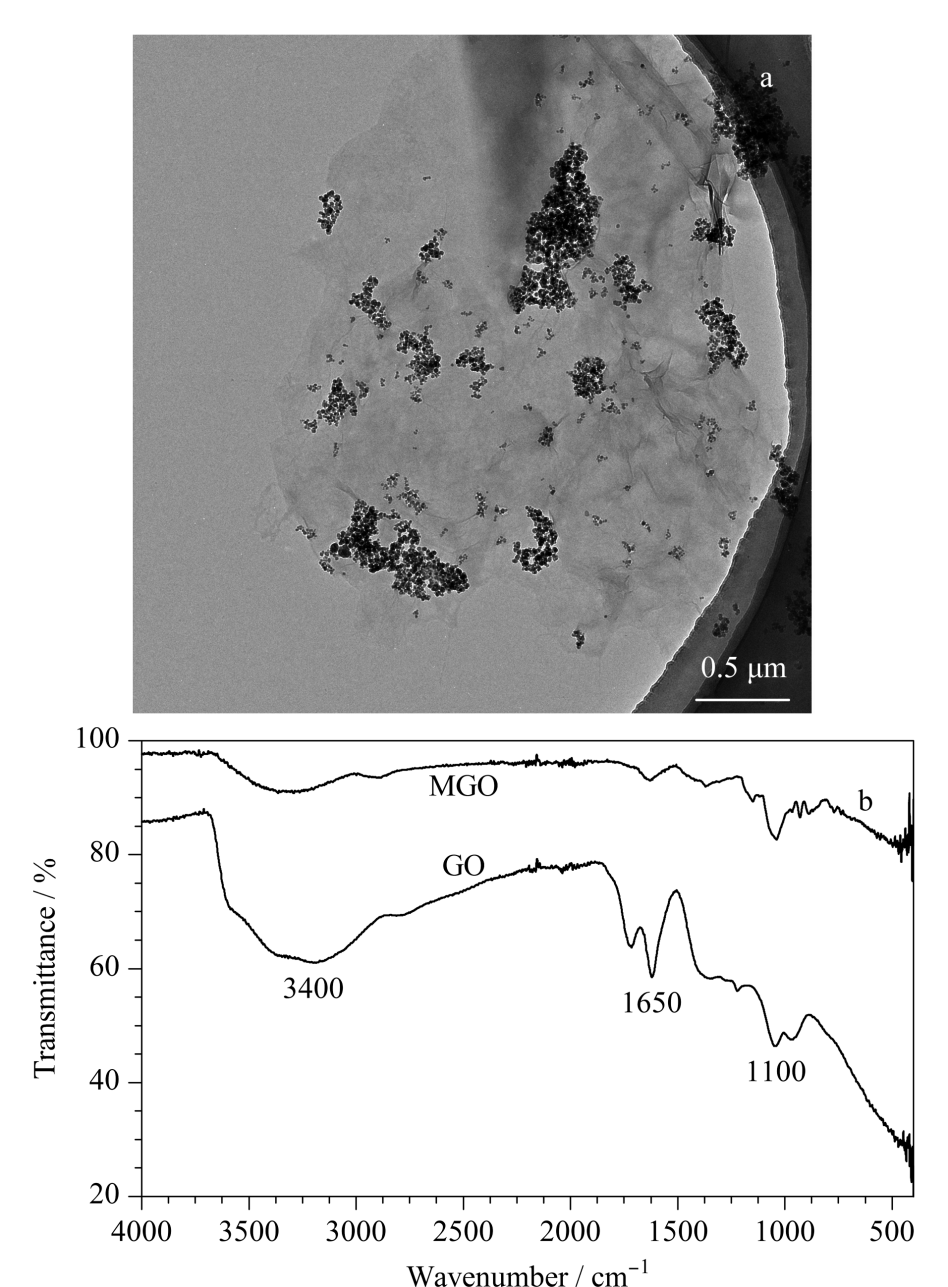
(a)MGO的TEM图,(b) MGO和GO的傅里叶变换红外光谱图

### 2.2 MGO-FAM-Apt传感器检测E2

基于上述MGO材料,我们构建了MGO-FAM-Apt传感器,其原理基于GO与FAM-Apt产生的荧光共振能量转移(FRET)效应^[26]^。如图2a所示:在没有目标检测物E2存在时,MGO通过*π-π*堆积与FAM-Apt作用,发生FRET效应,能量从供体FAM-Apt转移到受体MGO,从而猝灭荧光;而当有E2存在时,E2与FAM-Apt形成复合物^[18]^,使FAM-Apt从MGO上脱附下来,阻止FRET效应的发生,从而使荧光恢复。荧光信号的恢复程度与E2质量浓度在一定范围内成正比,因此构建了高灵敏检测E2的荧光生物传感器。

**图2:**
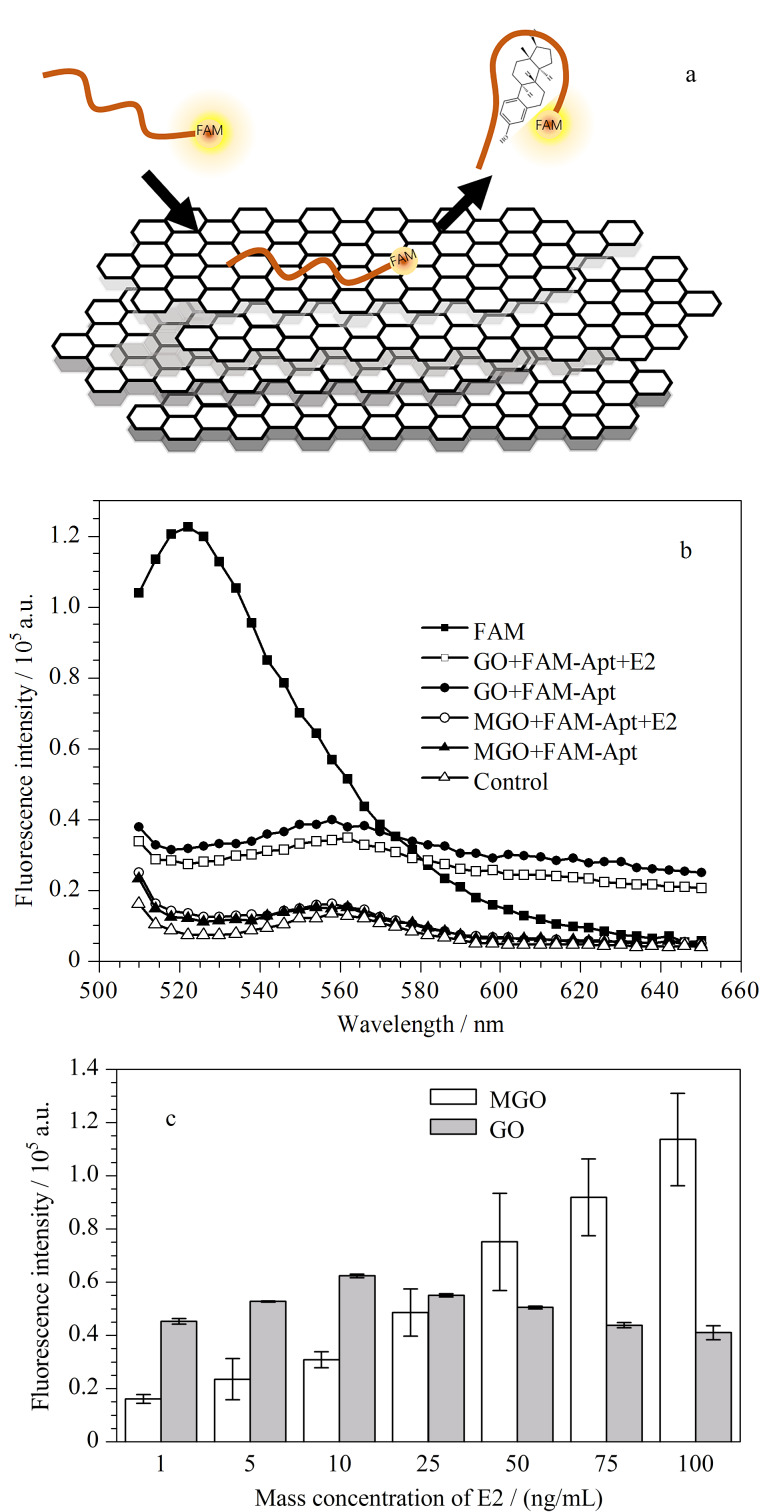
(a) MGO-FAM-Apt传感器检测原理示意图,(b)相同 反应体系下10 ng/mL E2恢复荧光的MGO、GO对比荧光光谱,(c)低浓度E2下,基于MGO和GO的传感器性能对比(*n*=3)

在我们所构建的体系中,在磁力架上分离MGO时,10 s内MGO可以完全吸附在管壁上,然后检测上清液中释放的FAM-Apt信号,实现对溶液中E2的分析。相比使用GO作为吸附剂时,MGO更易分离,且产生的背景荧光较低。如图2b所示,通过对比处理10 nmol/L的FAM-Apt后,以GO为吸附剂时的背景荧光值在520 nm处是以MGO为吸附剂时的两倍,这表明MGO对低浓度E2的荧光信噪比优于GO。采用基于MGO构建的E2检测方法具有操作简便、分析速度快和背景信号低的优点。我们对MGO对低浓度E2的检测效果进行了测试,在低质量浓度E2(1、5、10、25、50、75、100 ng/mL)的检测范围内,以MGO为吸附材料的E2荧光恢复值呈线性上升趋势,而以GO为吸附材料的传感器在不同E2浓度范围内的荧光值没有显著变化。这证明相较于GO, MGO材料对E2的检出限能够降低两个数量级。

### 2.3 MGO-FAM-Apt传感器性能优化

MGO-FAM-Apt传感器的设计基于MGO吸附并猝灭FAM-Apt荧光以及E2与FAM-Apt结合这两个关键过程。为了提高检测效果,需要优化E2与FAM-Apt复合物的稳定性以及MGO对游离FAM-Apt的吸附量,主要影响因素包括MGO的吸附量、pH值和盐浓度。首先考察了MGO用量对传感器吸附效果的影响。图3a显示,当体系中FAM-Apt终浓度为10 nmol/L时,在200 μL的反应体系中加入20 μL的MGO时猝灭效果最佳。进一步研究了pH值的影响,图3b表明在pH等于9时荧光恢复强度最高,可能是因为pH对FAM荧光强度的影响占主导地位。由于FAM的荧光强度受pH值影响,在偏碱性条件下荧光较强,因此最终确定了缓冲体系的pH为9。最后,盐浓度是另一个重要因素,包括镁离子和钠离子浓度。这两者不仅影响MGO的吸附效果,还通过改变ssDNA的二级结构影响E2-FAM-Apt复合物的稳定性。图3c、3d显示,当镁离子浓度低于5 mmol/L时,背景荧光值较高且MGO的吸附效果较差。然而,当镁离子浓度高于5 mmol/L时,E2荧光恢复值随着镁离子浓度的增加而降低,因此最佳镁离子浓度为5 mmol/L。钠离子浓度在不大于100 mmol/L时,荧光恢复值与钠离子浓度呈正相关;而当浓度高于100 mmol/L时,荧光恢复值趋于稳定。因此,最佳钠离子浓度确定为100 mmol/L。

**图3:**
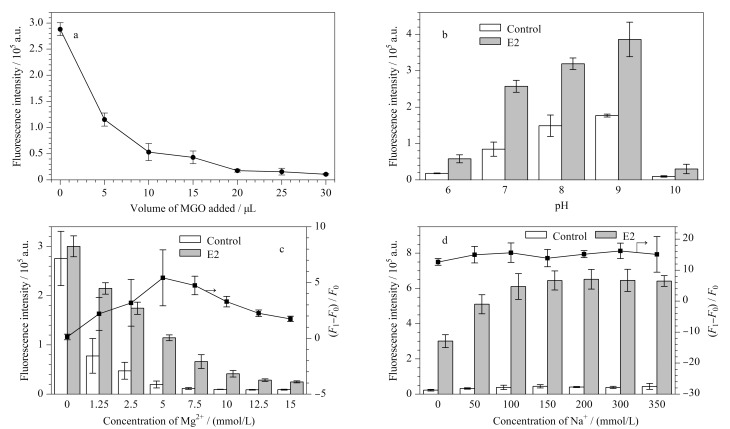
(a) MGO加入量、(b) pH、(c)镁离子浓度及(d)钠离子浓度的优化(*n*=3)

### 2.4 抗干扰能力与特异性评价

由于水样可能含有钙离子、铁离子、锰离子、硫酸根和硝酸根等干扰离子,我们测试了这些不同干扰离子对传感器检测的影响。实验结果显示,钙离子、锰离子、硫酸根和硝酸根对E2的检测影响较小,但加入0.3 mg/L的铁离子会略微降低E2的荧光恢复强度。鉴于传感器中FAM-Apt与E2的结合是特异的,我们引入了相同浓度的4种雌激素类干扰物——E3、EE2、E1和BPA,来评估传感器对E2检测的特异性。实验结果显示,在相同的反应条件下,E2的恢复荧光信号值最高,而E1和E3能够部分恢复荧光信号,其中E1的相对荧光信号值为E2的33%, E3的相对荧光信号值为E2的23%,而EE2和BPA几乎不能恢复荧光信号。这表明我们所构建的MGO-FAM-Apt传感器对E2的检测具有特异性,雌激素类似物的干扰较小。

**图4:**
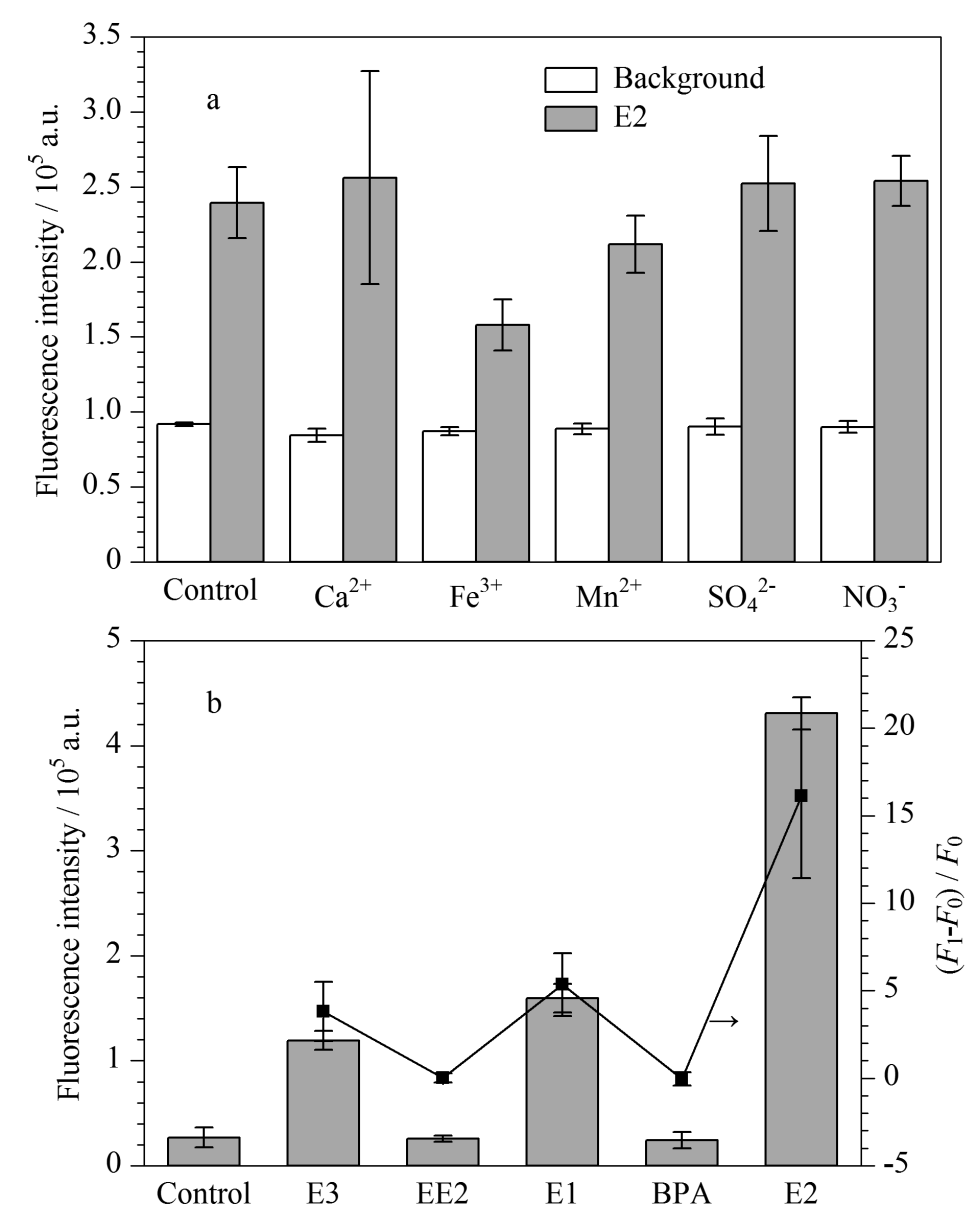
(a)不同干扰离子对检测10 ng/mL E2的影响,(b) MGO-FAM-Apt传感器的特异性(*n*=3)

### 2.5 与LC-HRMS方法的分析性能比较

我们使用了Ma等^[27]^建立的LC-HRMS方法对一系列E2样品进行了测定。E2样品以甲醇溶解,并通过Accucore Vanquish C18柱(50 mm×2.1 mm, 1.5 μm, Thermo Fisher Science)进行分离。流动相为超纯水和甲醇,流速为0.3 mL/min。质谱仪在正负电喷雾电离模式下运行,采用全扫描模式获取目标分析的质谱图信号。E2母离子*m/z*为271.21,子离子*m/z*分别为154.21和183.18。图5a显示,在对两组离子对(*m/z* 271.21/154.21和*m/z* 271.21/183.18)进行定量分析后,在保留时间为7.12 min处的信号强度与E2质量浓度呈线性相关。使用E2标准溶液质量浓度分别为0.5、1、2、5、10、20、50、100、200、500、1000 ng/mL绘制了标准曲线,线性方程为*y*=3470*x*+28972(*R*^2^=0.997),检出限为0.2 ng/mL。根据图5a的结果,可以观察到0.1 ng/mL和0.2 ng/mL的响应信号几乎没有区分度,因此我们确定了定量限为0.5 ng/mL。在0.5~1000 ng/mL范围内,质谱响应信号与E2质量浓度呈线性关系。

**图5:**
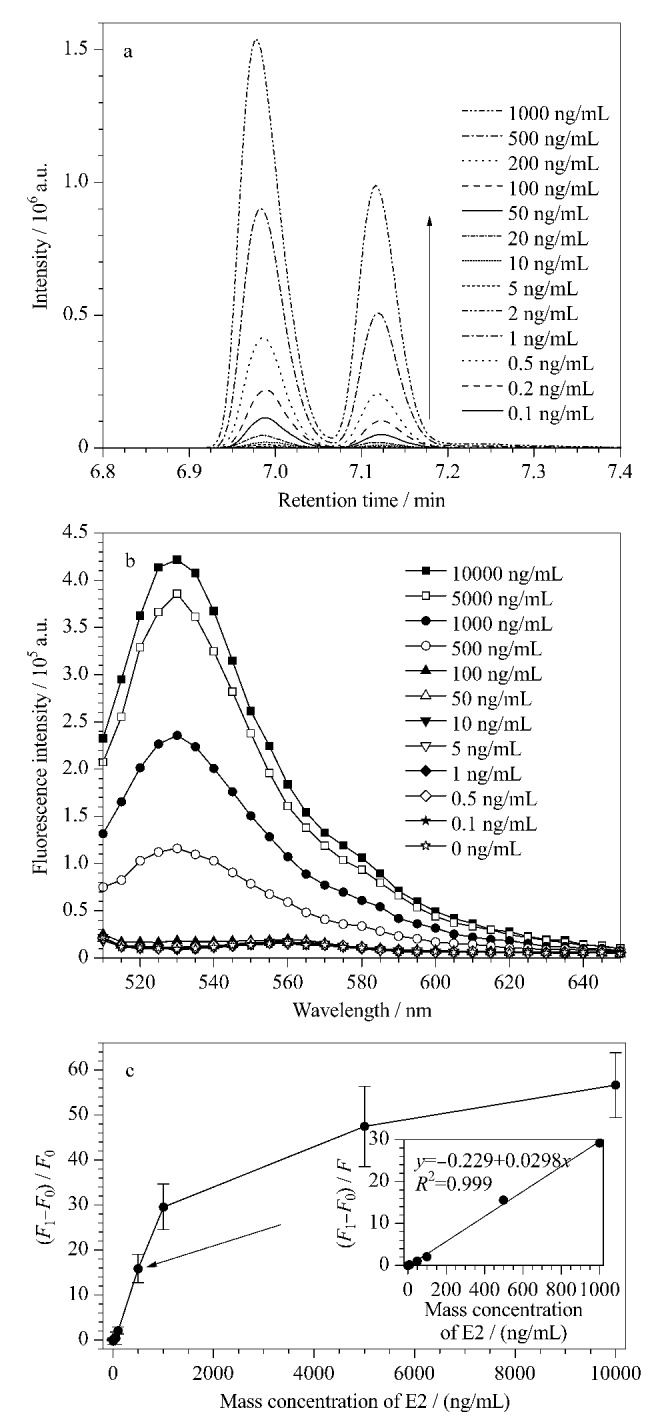
(a) LC-HRMS方法分析E2的色谱图,(b) MGO-FAM-Apt传感器分析E2的荧光光谱图,(c) MGO-FAM-Apt传感器的荧光响应-质量浓度图(*n*=3)

为了评估所构建的传感器对E2的检测性能,我们在最佳反应条件下测定了不同质量浓度E2所产生的荧光恢复信号,其荧光光谱图如图5b所示。根据图5b的结果,我们绘制了相对荧光恢复值-E2质量浓度曲线,如图5c所示,可以观察到在1~1000 ng/mL范围内,相对荧光恢复值随着E2质量浓度的增加而增加,呈现良好的线性关系,检出限为1 ng/mL;超出1000 ng/mL后相对荧光恢复值逐渐达到平台期,超出检测范围。相较于LC-HRMS方法,尽管MGO-FAM-Apt传感器方法在检出限方面仍存在差距,但其具备低成本、短检测时间、可同时检测多个样品等优势。因此,该方法可用于现场检测或高通量样品检测。

将本方法与近年来发展的其他基于适配体的E2检测方法相比,如表1所示,本方法对E2检测的线性范围宽,检出限低。

**表1:** 基于适配体的不同E2检测方法与本方法的对比

Method	Linear range		LOD/ (ng/mL)	Time/ min	Ref.
Fluorescence	50-800	ng/mL	34.5	/	[11]
Fluorescence	5-75	nmol/L	0.57	10	[16]
Fluorescence	80-400	nmol/L	10	60	[13]
ELISA	1.5-50	nmol/L	0.41	/	[14]
Colorimetric	6.7-66700	nmol/L	1.8	50	[12]
Colorimetric	1.57-350	nmol/L	0.44	55	[17]
Colorimetric	/		136	120	[15]
Fluorescence	1-1000	ng/mL	1.0	15	this work

ELISA: enzyme-linked immunosorbent assay. /: data not mentioned.

### 2.6 实际样品分析

我们选择了清河水样进行实际样品分析。为了评估MGO-FAM-Apt传感器的性能,我们对水样进行了加标试验。由于环境水样中E2的浓度通常较低,因此我们进行了3个较低浓度的加标试验。实验结果显示,在低质量浓度范围(1~10 ng/mL)内,MGO-FAM-Apt传感器的加标回收率为91.0%~110.0%(见表2),表明我们所构建的MGO-FAM-Apt传感器可应用于环境水样中E2的检测。

**表2:** 水样中E2在3个水平下的加标回收率(*n*=3)

Added/(ng/mL)	Found/(ng/mL)	Recovery/%	RSD/%
1	1.10	110.0	7.7
	1.02	102.0	
	0.91	91.0	
3	3.13	104.3	2.3
	3.07	102.3	
	2.96	98.7	
6	6.03	100.5	0.57
	6.04	100.7	
	6.11	101.8	

## 3 总结

本研究采用改进的微乳液合成法,将Fe_3_O_4_纳米磁珠包覆在GO上,制备了MGO。通过MGO与核酸适配体之间的相互作用,构建了一种“turn-on”型MGO-FAM-Apt荧光生物传感器。该传感器具有核酸吸附性能强和荧光猝灭效率高的特点,能够用于环境样品中低浓度E2的检测,具有成本低、检测快速、选择性和灵敏度良好等优点。尽管与传统的色谱-质谱法相比,在检出限方面仍存在差距,但它可以作为一种方便、快速的现场检测方法,用于环境样品的高通量筛查,为解决环境中17*β*-雌二醇污染问题提供了一种新的选择。
